# The senescence journey in cancer immunoediting

**DOI:** 10.1186/s12943-024-01973-5

**Published:** 2024-04-01

**Authors:** Alessandra Zingoni, Fabrizio Antonangeli, Silvano Sozzani, Angela Santoni, Marco Cippitelli, Alessandra Soriani

**Affiliations:** 1 Department of Molecular Medicine, Laboratory Affiliated to Istituto Pasteur Italia-Fondazione Cenci Bolognetti, Sapienza University of Rome, Rome, 00161 Italy; 2 Institute of Molecular Biology and Pathology, National Research Council (CNR), Rome, 00185 Italy; 3 IRCCS Neuromed, Pozzilli, 86077 Italy

**Keywords:** Cancer, Senescence, SASP, Immunoediting, Immunosurveillance, Dormancy, Senotherapy

## Abstract

Cancer progression is continuously controlled by the immune system which can identify and destroy nascent tumor cells or inhibit metastatic spreading. However, the immune system and its deregulated activity in the tumor microenvironment can also promote tumor progression favoring the outgrowth of cancers capable of escaping immune control, in a process termed cancer immunoediting. This process, which has been classified into three phases, i.e. “elimination”, “equilibrium” and “escape”, is influenced by several cancer- and microenvironment-dependent factors. Senescence is a cellular program primed by cells in response to different pathophysiological stimuli, which is based on long-lasting cell cycle arrest and the secretion of numerous bioactive and inflammatory molecules. Because of this, cellular senescence is a potent immunomodulatory factor promptly recruiting immune cells and actively promoting tissue remodeling. In the context of cancer, these functions can lead to both cancer immunosurveillance and immunosuppression. In this review, the authors will discuss the role of senescence in cancer immunoediting, highlighting its context- and timing-dependent effects on the different three phases, describing how senescent cells promote immune cell recruitment for cancer cell elimination or sustain tumor microenvironment inflammation for immune escape. A potential contribution of senescent cells in cancer dormancy, as a mechanism of therapy resistance and cancer relapse, will be discussed with the final objective to unravel the immunotherapeutic implications of senescence modulation in cancer.

## Background

There are several factors influencing tumor progression, including genetic alterations, epigenetic modifications, oncogenic signals, and inflammatory cytokines. Among these, the contribution of the immune response on cancer cell fate is crucial, playing a dual role potentially limiting or, paradoxically, promoting tumor growth [1, 2].

In 1909, Paul Ehrlich postulated the concept that the immune system can recognize and eliminate cancer cells (Ned Tijdschr Geneeskd. 1909;5:273–290) and in the late 1950s Burnet and Thomas further expanded this concept with the immunosurveillance theory of cancer, hypothesizing that lymphocytes act as sentinels capable of recognizing and eliminating nascent tumor cells before they manifested disease [3] (L Thomas, H Lawrence - New York: Hoeber-Harper, 1959 - IET). However, the concept of cancer immunosurveillance as currently understood, was developed by Robert Schreiber who formulated the theory of cancer immunoediting [1]. This not only incorporates the original notion of cancer immunosurveillance but also recognizes that even after immunosurveillance escape, a tumor phenotype is continuously sculpted by the immune system. According to this view, cancer immunoediting is a multi-step process consisting in three phases: “Elimination”, “Equilibrium” and “Escape”, also referred to as the three “Es”.

Considering the numerous biological and genetic mechanisms that regulate aging, herein we focus on one of the key processes of aging — cellular senescence — which links aging and cancer together. Senescent cells (SnCs) accumulate during aging, contributing to tissue dysfunction and different pathological conditions, as well as playing a role in tumorigenesis and cancer progression. It has been described that the elimination of SnCs can potentially reduce age-related pathologies, including cancer [4]. In healthy individuals, activation of the immune system can rapidly clear SnC accumulation following induction, strongly decreasing their detrimental activity in tissues [4, 5]. However, during cancer progression or in older individuals, this control is less efficient or partially inhibited, leading to the accumulation of SnCs [6]. The impact of senescence in the regulation of anti-cancer immune responses is only beginning to be elucidated. In this regard, induction of senescence has been correlated with tumor suppression by inhibiting tumorigenesis of pre-neoplastic cells. On the other hand, SnCs have also been implicated as an active driving force to cancer progression, by sustaining immune-related hallmarks of cancer, such as inflammation and immune evasion. In this review, we will discuss how senescence, in response to various intrinsic and extrinsic stimuli, can regulate cancer development through a journey in the different steps of cancer immunoediting.

## Senescence at a glance: definition, markers and SASP

**Fig. 1:**
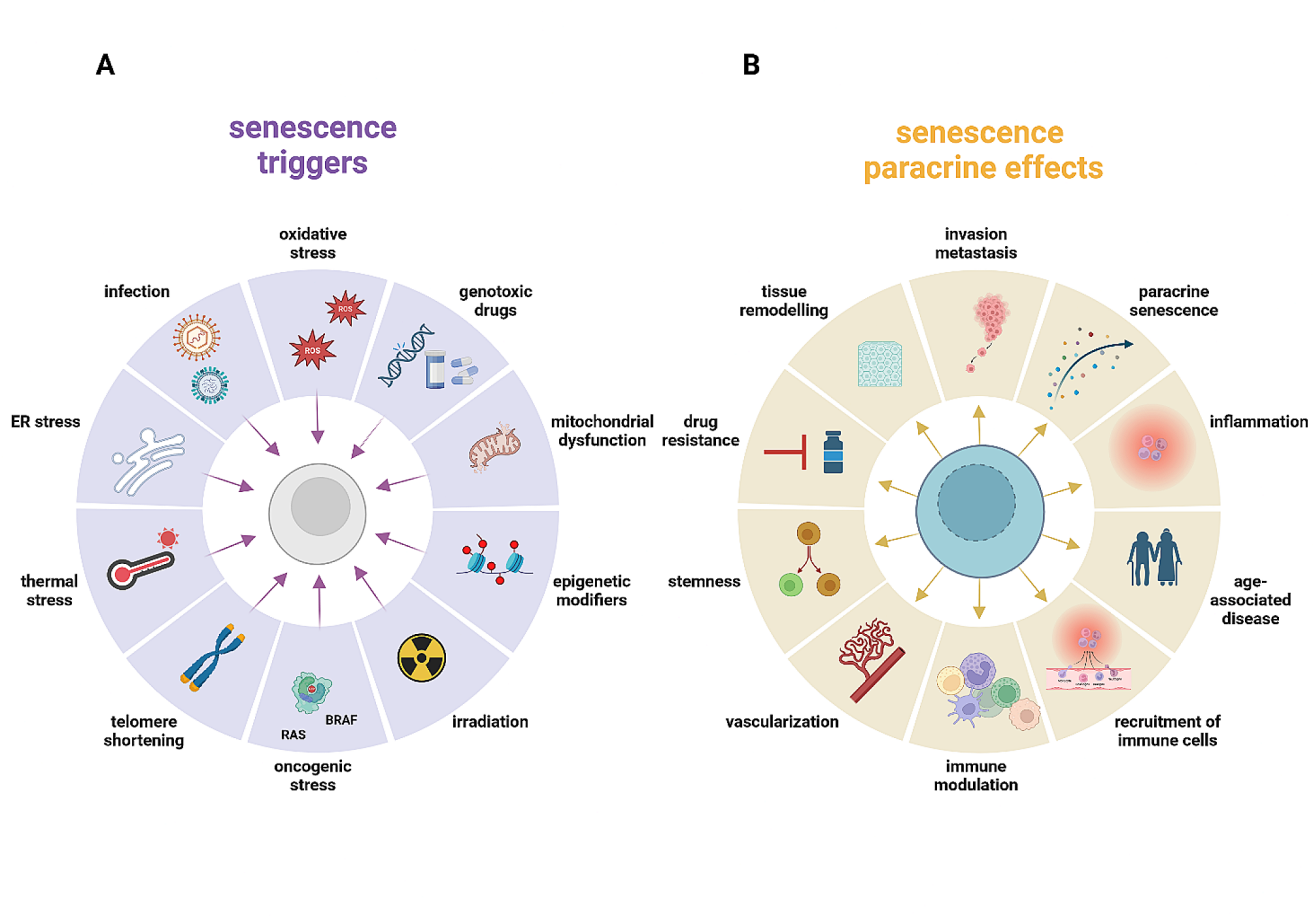
Senescence inducers and paracrine effects of SnCs Overview of senescence inducers and paracrine effects of SnCs on the surrounding microenvironment. The senescence program can be activated by different stress stimuli (shown on the left) such as cytotoxic chemotherapeutic drugs, replicative and oxidative stress. On the other hand, SASP factors produce paracrine effects impinging on cancer onset and progression (shown on the right). SASP can induce senescence of surrounding cells (paracrine senescence), facilitate tissue remodeling and recruitment of immune cells Created with BioRender.com with granted permission and license

The word senescence derives from the Latin word “senex” meaning “growing old”. Indeed, senescence was originally described in 1961 as the cellular program based on cell cycle arrest distinguishing human diploid fibroblasts after long-term in vitro culture [7]. Today, senescence is seen as a hallmark of aging and cancer [8, 9]. Cells undergoing senescence display distinctive morphological and functional alterations, but without unique molecular markers. They are characterized by a deep proliferative arrest (upregulation of cyclin-dependent kinase inhibitors p16 and/or p21), changes in cell morphology (size enlargement and perinuclear lamin-B1 downmodulation), epigenetic modifications (senescence-associated heterochromatin foci), and activation of specific signaling pathways (DNA damage response (DDR) and PI3K/FOXO/mTOR). The expansion of the lysosomal compartment accounts for the senescence-associated beta-galactosidase (SA-β-Gal) activity and represents the most reliable cellular marker of senescence along with lipofuscin accumulation [10]. Throughout life, several intrinsic and extrinsic injuries, via different pathways, trigger senescence (Fig. 1A).

Oncogene-induced senescence (OIS) following aberrant hyperproliferation, and chemotherapy- and radiotherapy- induced senescence (TIS), have long been described as key components in tumor biology [11]. However, how and to what extent cellular senescence affects cancer is still unclear. SnCs exhibit robust metabolic reprogramming, leading to a peculiar senescence-associated secretory phenotype (SASP), which consists of the release of bioactive molecules and inflammatory factors into the surrounding microenvironment resulting in immune cell recruitment and orchestration of tissue homeostasis [12] (Fig. 1B). It is reasonable to assume that senescence developed as a cellular stress response to disarm and shut down cell division, in due course removing damaged cells by alerting the immune system [13].

SASP composition is heterogeneous and highly dynamic. It is cell-type specific, trigger-dependent and changes over time, with successive stages of factor secretion. Nevertheless, a core group of molecules can be identified by an overlapping presence of Angiogenin, CCL2, GM-CSF, GRO-α, -β, -γ, ICAM-1, IGFBPs, IL-1, IL-6, CXCL8, MMP-1, MMP-3, MMP-10, Osteoprotegerin [14]. Recently the extracellular vesicles have been included as key mediators within SASP [15] and contribute to modulate the phenotype of recipient cells resulting in inflammation, cancer and immune cell modulation, and paracrine senescence induction [16–22].

Collectively, these factors characterize a troop of SnCs aimed at tissue remodeling, bolstered through the immune system, with inevitable repercussions on tumor progression. An additional layer of complexity is also related to the induction of a stromal SASP that can interact with tumor cells and may be involved in tumor initiation and progression, although how it potentially affects immune cells is still unclear [23–25].

The NF-kB and C/EBPβ transcription factors are the main drivers for the expression of SASP-related genes, with the transcription factor GATA4 also implicated [26]. In addition, the discovery that SnCs lose nuclear envelope integrity, leading to the production of cytoplasmic chromatin fragments (CCFs), links SASP induction to the cGAS-STING pathway as it will be extensively discussed in a separate section below.

## Senescence-driven immune surveillance: the “elimination” phase

**Fig. 2:**
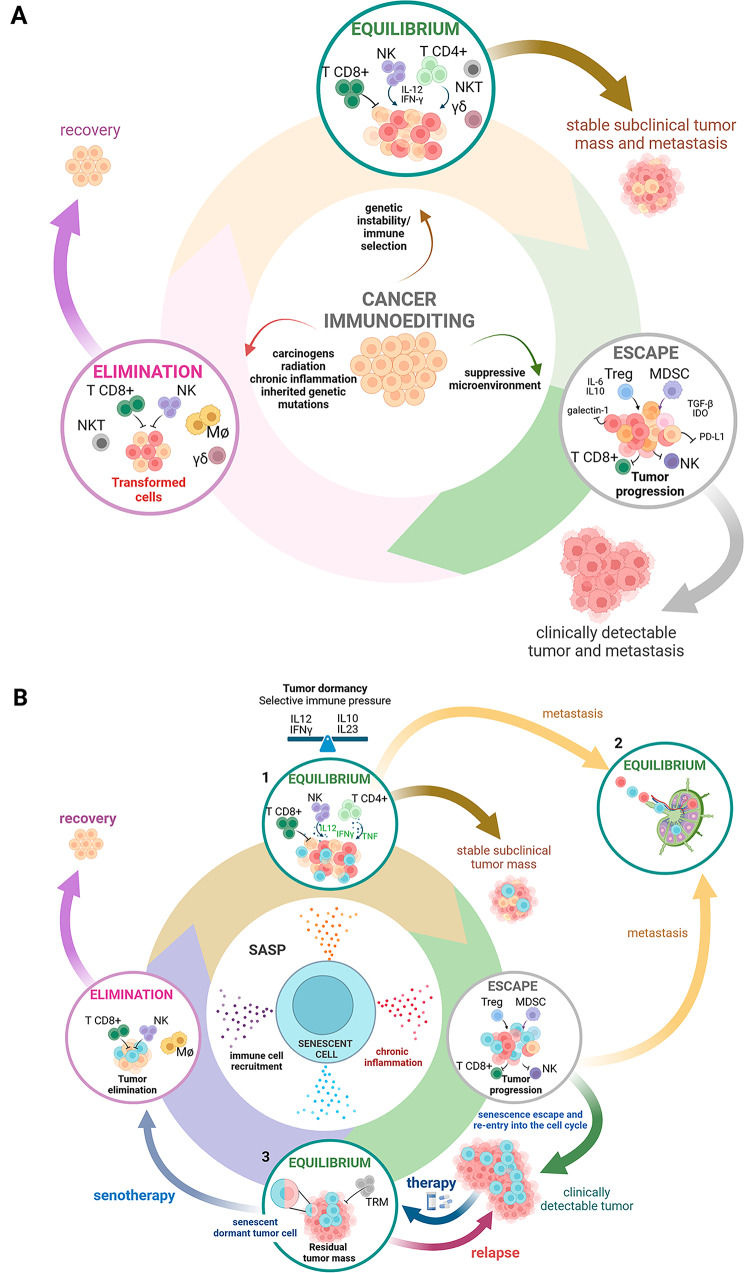
SASP into the different steps of cancer immunoediting (**A**) Cancer immunoediting is a dynamic process between tumor cells and the immune system and it has been outlined in the 3Es. In the first phase of elimination, innate and adaptive immunity destroys developing tumors before they become clinically evident. If this step is performed successfully the tumor cells are removed. If, however, the tumor cell variants are not destroyed, the equilibrium phase can be entered, in which tumor growth is confined by immunological mechanisms in a state of functional dormancy. In this phase tumor cell variants may emerge as a consequence of constant immune selection pressure and may enter into the escape phase, in which their growth is no longer blocked by immunity and tumor becomes clinically detectable. (**B**) *SASP* can lead to the clearance or the protection of cancer cells, favoring cell dormancy and tumor recurrence. In the “elimination phase” SnCs are efficiently eliminated by both innate and adaptive immune cells. Initially SASP released by oncogene-induced senescent tumor cells can recruit immune cells into the tumor bed favoring the elimination of cancer cells. During this phase some cancer cells may remain and contribute to the establishment of an equilibrium between the tumor and the immune system. In this “equilibrium phase 1”, the immune system holds the tumor in a state of functional dormancy and senescent cells may contribute by releasing a plethora of different factors conditioning the TME. During this phase there is a balance between anti-tumor and tumor promoting cytokines and due to constant immune pressure, tumor cell variants evolve that resist immune recognition and drive the following “escape phase”. As a further layer of complexity some variants that have acquired the ability to metastasize leave the primary tumor (“equilibrium phase 2”), and senescent tumor cells can be found within metastatic lesions. In the “escape phase” senescent tumor cells release a pro-inflammatory SASP and may re-entry into the cell cycle contributing to a clinically detectable tumor that can be treated by therapy. Following treatment, a new “equilibrium phase 3” can be achieved in which it is possible to observe a residual tumor mass. The final use of senotherapy can lead to complete recovery. Abbreviation: TRM (Tissue resident memory cells); MDSC (Myeloid-derived suppressor cells) Created with BioRender.com with granted permission and license

The elimination phase is classically regarded as a bidirectional crosstalk between pre-malignant/malignant cells and cells of the immune system in which the pressure of the immune system is successful at halting cancer cells (Fig. 2A). This phase is considered largely dependent on the mutational burden of tumor cells, with the expression of tumor neoantigens (TNAs). Much relevance is given to the immunogenicity of TNAs and its associated immune surveillance (be it natural or therapy-elicited) [27]. This straightforward view is now moving towards a more complex picture, in which different cancer cell phenotypes, such as stem- and dormant-like phenotypes, along with several elements of the tumor microenvironment (TME) heterogeneously affect antitumor innate and adaptive immunity. In this scenario, cellular senescence has recently been proposed as an emerging hallmark of cancer [9] and it acts as a double-edged sword showing opposing effects on tumor progression [28]. Regarding its contribution to the elimination of cancer cells, it should be noted that the senescent phenotype can occur at different times and in different cell types of tumor stages. OIS characterizes premalignant cells and is a major barrier to tumorigenesis. It restrains tumor growth by arresting the proliferation of cells at risk of malignant transformation and, at the same time, promotes the immune-mediated clearance of dangerous cells. TIS is an alternative cellular fate to other forms of regulated cell death triggered by conventional chemotherapy, radiation, and molecularly targeted agents. Thus, TIS commonly comes when cancer is clinically evident, a stage of tumor usually ascribed to the equilibrium or escape phases. Moreover, TIS can affect cancer cells as well as cells of the TME, such as stromal and endothelial cells, with different biological outcomes. The impact of these senescence-related temporal and spatial differences is not fully understood. Data from literature support the notion that cellular senescence can promote the elimination phase by at least three mechanisms: (i) immune cell recruitment; (ii) direct killing enhancement; (iii) antigenicity/adjuvanticity properties.

i) *immune cell recruitment.* The first evidence of a role of senescence to cancer immunosurveillance through immune cell recruitment came from groundbreaking work by Scott Lowe and colleagues. By treating lymphoma with cyclophosphamide, a drug usually reported to induce apoptosis, they showed that cellular senescence developed in vivo following chemotherapy [28]. Moving on, investigating a genetically modified mouse model, they provided direct evidence that cellular senescence triggered by p53 restoration in liver carcinoma induced by the oncogenic H-RAS expression, resulted in tumor regression due to an innate immune response against senescent tumor cells. This response was guided by macrophages and Natural Killer (NK) cells migrated into the tumor bed following the release of CCL2 (previously called MCP1), CXCL1, and IL-15 [29]. Similar findings were reported by others in sarcomas [30]. Thus, p53 controls molecular programs central for senescence, whilst complementary pathways mainly regulated by NF-κB contributes to the SASP, which provides a plethora of inflammatory factors e.g., IL-6, CXCL8, CXCL1, and ICAM1 necessary for the recruitment and activation of immune cells [31]. Importantly, wild-type p53 has been described to interact with NF-κB both in a cooperative and also antagonistic manner [32]. The final outcome can be context-dependent and modulated by factors such as IKKα activity [33] or the common TP53 codon 72 polymorphism [34]. In this scenario, it is not surprising that the alterations of p53 activity caused by different TP53 mutations can result in a dysregulated senescence and tumor secretome, affecting the complex crosstalk between tumor cells and a plethora of cell types in the surrounding stromal microenvironment. Indeed, mutant p53 activities are often dependent on other transcription factors expressed (or activated) in a stimulus and/or cell-specific manner (reviewed in [35]). In this regard, a shift of the secretome from a tumor suppressive (driven by wt-p53) to a cancer-promoting one has been described in models of p53 loss of function (LOF), dominant-negative (DN) inhibition by mutant p53 or gain of neomorphic functions (GOF) [36–38].

In a different model, the SASP of N-RAS-dependent pre-malignant senescent hepatocytes, which supplies CCL2 and IL-1α, is sufficient to drive an immune response that limits liver cancer development. In particular, CCR2^+^ myeloid cells are recruited into the tumor bed and a CD4^+^ T cell-mediated response stimulates macrophages to clear pre-malignant SnCs [39, 40]. In this way, OIS effectively contributes to the elimination phase by alerting both the innate and adaptive arms of the immune system. Moreover, CCL2 plays a key role in the elimination of senescent cancer cells in liver carcinoma mostly by driving NK cell recruitment to the tumor [41]. Accordingly, the abrogation of SASP gene expression by bromo and extra terminal domain (BET) protein inhibitors, leads to the loss of immune-mediated cancer surveillance [42]. In senescent nevi prone to melanoma transformation, senescence induction by epigenetic drugs is associated with a considerable increase of melanoma infiltrating CD11b^+^ innate immune cells [43]. Collectively, these studies have changed the understanding of senescence, from a cell-autonomous (intrinsic) defense mechanism restraining cell proliferation, towards an extrinsic tumor barrier involving the action of the immune system.

The ability of senescence in recruiting immune cells to tumors underpins the clinical success of different cytostatic agents adopted in cancer therapy. A combined treatment with a MEK inhibitor (trametinib) and a CDK4/6 inhibitor (palbociclib) in K-RAS-mutant lung and pancreatic cancer cells was able to induce cellular senescence with cytotoxic effects in vivo due to the recruitment (via CCL2, CCL4, CCL5, CXCL10, CX_3_CL1) and activation (via IL-15, IL-18, TNF-α) of immune cells, namely NK cells [44]. TIS can also sustain SASP-mediated vascular remodeling in pancreatic ductal adenocarcinoma with the result of improving both drug delivery and lymphocyte infiltration to the tumor core, leading to windows of opportunities for enhancing susceptibility to otherwise ineffective chemo- and immuno-therapies [45]. Hence, TIS can revert the equilibrium or escape phases by restoring the elimination phase.

ii) *direct killing enhancement.* The immune clearance of SnCs, a process called “senescence surveillance”, is of great relevance to tumor progression as the life span of SnCs in tissues deeply affects the inflammatory processes. Different cellular types are implicated in the immunosurveillance of SnCs, with macrophages and NK cells the most involved (Fig. 2). In a model of liver carcinogenesis by CCl_4_ fibrogenic treatment with coadministration of the carcinogen diethylnitrosamine, p53-dependent senescence skewed macrophage polarization towards an “M1-like” phenotype with SnC-targeting capacity [36]. In a different approach designed to identify unique biomarkers of senescence, senescent primary human fibroblasts were found to expose an oxidized form of membrane-bound vimentin (i.e., malondialdehyde-modified vimentin), specifically recognized by a natural polyreactive IgM antibody that likely enables phagocytosis of SnCs by macrophages [46]. Regarding the mechanisms of direct killing activity, NK cells have been studied in more detail [47]. The ability of NK cells to target SnCs is mediated by the activation of the NKG2D and DNAM-1 receptors. The corresponding stress-induced ligands are strongly upregulated by SnCs following both OIS and TIS [5]. Efficient killing of SnCs by NK cells requires granule exocytosis and not death receptor signaling [48]. Notably, NKG2D and DNAM-1 engagement also triggers the production of IFN-γ that might boost macrophage activity, possibly aiding the clearance of SnCs [49]. Senescent tumor cell clearance by NK cells has been thoroughly investigated in the context of multiple myeloma by our group. Myeloma cells treated with low doses of therapeutic agents commonly used in the management of patients with multiple myeloma, such as doxorubicin, melphalan, and bortezomib, up-regulate NK cell-activating ligands for the NKG2D and DNAM-1 receptors, increasing NK cell degranulation in vitro and in vivo, an effect preferentially associated with SnCs arrested in the G2 phase of the cell cycle [50, 51]. Genotoxic agents initiate a redox-dependent DDR that increases MICA and PVR NK cell activating ligands on senescent multiple myeloma cells through the transcriptional factor E2F1, indicating the immunogenic senescence as a key mechanism promoting the clearance of drug-treated tumor cells by innate effector lymphocytes [49]. Moreover, drug-induced senescent multiple myeloma cells can cross-present IL-15/IL-15RA complex on their membrane, thus stimulating activation and proliferation of NK cells [22].

NK-mediated cytotoxicity has also been attributed to the expression of ICAM-1 on lung tumor cells following TIS [44] and to direct intercellular CDC42 protein transfer from SnCs to NK cells in the preneoplastic pancreas, which leads to actin rearrangement and thus NK cell activation [52].

iii) *antigenicity/adjuvanticity properties.* As previously outlined, senescent hepatocytes expressing MHC class II and CD86 mediate CD4^+^ T lymphocyte activation, stimulating a macrophage response against pre-malignant senescent hepatocytes [39]. In this context, recent findings highlighted the specific contribution of the adaptive immune system. By investigating different models of senescence (drug-induced, p53-driven in fibroblasts, melanoma, and pancreatic adenocarcinoma), Serrano and colleagues demonstrated a type I IFN-dependent upregulation of MHC-I molecules on SnCs, along with an elevated senescence-associated epitope presentation. This led to CD8^+^ T lymphocyte activation as revealed by CD25 and CD69 expression. In addition, alarmins (ATP, calreticulin) released from SnCs were accompanied by greater dendritic cell maturation (MHC-II, CD80, and CD86 expression) than that observed in the immunogenic cell death. Remarkably, autologous antigen-specific tumor infiltrating lymphocytes were restimulated by senescence induction [53]. In the model of hepatocyte transformation by oncogenic RAS and senescence induction by p53 reactivation, Scott Lowe et al. reported complementary findings. The authors found a stark switch from an immunosuppressive tumor environment characterized by exhausted T lymphocytes to an immunocompetent setting with functional CD8^+^ T lymphocytes and active macrophages following senescence induction attributing these changes to a cell-surface proteome remodeling of SnCs, which includes the increase of antigen-presenting machinery and heightened responsiveness to IFN-γ [54]. Taken together, these studies emphasize that senescence potently enhances the immunogenicity of cancer cells and supports the pioneering work of Weichselbaum and colleagues that suggests to use SnCs as cancer vaccination agents [55], as for the loading of dendritic cell-based vaccines. Furthermore, the finding that senescence acts as an immunostimulatory response to drugs better than the immunogenic cell death gives rise to the possibility of dosage lowering, with the aim of reducing side effects. The identification of senescence-specific antigens in cancer cells leads to hypothesize the existence of senescence-associated antigens in senescent aged tissues with the possibility of tailoring anti-aging therapies. Molecular details on the immunopeptidome generation are still missing, but a fascinating idea is a possible link with the enhanced mobilization of retrotransposable elements that characterizes senescent cells and the following cGAS-STING pathway stimulation that in turn supports the MHC I machinery [56, 57]. As addressed below in a separate section, upregulation of the inhibitory molecule PD-L1 and PD-L2 can also occur on SnCs and this accounts for the failure of anti-tumor immunity [58]. The overall immunogenicity of a senescent tumor cell is thus a fine balance that reflects the intrinsic history of the single cell, its surrounding milieu in term of cytokines and chemokines and the compartmentalization of infiltrating immune cells in the tumor.

## Senescence within the “equilibrium phase”

In cancer immunoediting, the “equilibrium phase” is when the expansion of transformed cells is arrested and maintained in a state of functional dormancy by immunity. If during the “elimination phase” the innate and adaptive immune system is unable to completely destroy the tumor, it converts cancer cells in a quiescent mass, resulting in a state of equilibrium between the developing tumor and the action of the immune system, which may occur over a period of years. In this phase the tumor dormancy is specifically controlled by adaptive immunity [59, 60] (Fig. 2A). Conceivably, the same immune functions that keep cancer cells in a “not expanding” status also provide the selective pressure responsible for the outgrowth of tumor cells once these cells have acquired immune evasive mutations.

Thus, the “equilibrium phase” is a condition that may occur before tumor development at the primary site (referred to as “equilibrium 1”), after the spreading out of single cells at the metastatic sites referred to as “equilibrium 2”) or following therapies that lead to residual tumor mass (referred to as “equilibrium 3”) (Fig. 2B). Senescence and senescence-escape could serve as one mechanism of tumor dormancy and disease recurrence. Specifically, tumor cell senescence can represent a state of dormancy due to cell cycle arrest and apoptosis-resistance, whilst the SASP can establish a dormancy-promoting environment for “cancer stem cells”.

### Senescence as a form of in-depth cell dormancy

By definition, a ‘dormant cancer cell’ requires that the cell can reawaken and proliferate to repopulate a tumor. In this regard, while cellular senescence was initially believed to be irreversible, recent studies have shown that SnCs can re-enter the cell cycle in response to appropriate stimuli [61–65] and contribute to delayed relapse. This further supports the concept that senescence may represent one form of “in-depth dormancy”, whereby cancer cells evade the direct cytotoxic impact of therapy. Indeed, SnCs, like dormant tumor cells, are resistant to apoptosis in response to therapies that target proliferative cells. Unlike quiescent cells, they are unresponsive to growth factors and release paracrine signaling by the SASP that can impact the TME, thus sustaining delayed relapse and metastasis [66]. An additional point to consider concerning cancer cell dormancy is the accumulation of genetic and epigenetic changes selected in the tumor variants. This mechanism can leave a track of the immune system pressure [2]. Cell-cycle re-entry in the form of senescence escape may be a driver mechanism to interrupt this state of immune dormancy. Indeed, it is now clear that senescent tumor cells can resume proliferation through cell-autonomous and non-cell-autonomous mechanisms [43, 67–69]. Accordingly, a recent paper reveals that epigenetic memory defines post-SnCs in colorectal cancer, raising the possibility of early detection of lesions susceptible to cancer progression [70].

Reversible senescence has been mostly observed following TIS upon drug removal [63, 71–73]. Interestingly, endoreplication events leading to polyploidy are common after TIS [74] and there are studies pointing out that polyploidy may represent a prerequisite for succeeding in cell cycle re-entry [75, 76]. Indeed, senescent polyploid cells give rise to cycling euploid daughter cells by budding or asynchronous division [77]. It is conceivable that polyploidization supports the energetic and biosynthetic requirements of senescent cells from a transcriptional point of view. Furthermore, escaped cells often show more aggressive tumorigenic features, with increased stem-like markers or epithelial to mesenchymal transition traits [78, 79]. For comprehensive reviews on the topic see [80–82]. Mechanistically, senescence escape has been also linked to a lack of persistent DDR activation or not sustained mTOR signaling [83, 84]. Of course, it remains difficult to clearly distinguish between a regrowth from a locked cell cycle arrest (true senescence escape) or just a reprogramming from a not fully achieved senescent state (senescence bypass).

The concept that dormant cancer cells can remain dormant for many years suggests that tumor-specific immune memory acts as the key player in inducing and maintaining tumor dormancy via continuous durable pressure. In a model of T cell antigen-driven multistage carcinogenesis, Müller-Hermelink and colleagues found that specific Th1 cells were able to induce a state of tumor dormancy through a coordinated interaction between the cytokines IFN-γ and TNF [85]. Paradoxically, in the absence of both cytokines, the same T cells significantly promoted angiogenesis and multistage carcinogenesis [85]. In a different experimental setting, Th1 cell immunity arrested cancer progression through IFN-γ and TNF-induced senescence, the activation of p16INK4a, and downstream Rb hypo-phosphorylation at serine 795 [86]. Hence it is imperative to support endogenous anti-tumor immune responses through strategies aimed at reinvigorating antitumor immunity, or approaches capable of reversing tumor-induced immune tolerance [87, 88]. Of note, since IFN-γ and TNF induce senescence in numerous murine and human cancers, this may be a general mechanism for arresting cancer progression and maintaining senescent dormancy [89, 90].

The mechanisms by which dormant tumor cells preclude the recruitment or function of activated T cells remain largely unknown, but a leading hypothesis is that dormant cells persist likely due to poor interaction with endogenous antigen-specific T cells, a relatively rare population [91, 92]. However, in a model of triple-negative breast cancer (TNBC), despite close proximity to effector T cells, dormant tumor cells are not eliminated and support a CD4^+^ and FoxP3^+^ Treg-rich microenvironment. Secreted factors and DKK3, the Wnt pathway antagonist, were identified as a crucial mediators of this effect [93]. Additionally, immune evasion might be involved in cellular dormancy. As a matter of fact dormant leukemia cells express PD-L1, a mechanism of T-cell inhibition [94]. It is possible that senescent dormant cells may adopt similar escape mechanisms.

The outcome of the equilibrium phase depends on the balance between the strength of endogenous anti-tumor immunity and the tolerance mechanisms developed by tumor cells [2]. The frequency of various immune cell subsets among the tumor-infiltrating lymphocytes (TILs) from the dormant and progressive sarcomas have been analyzed in a model of induced mouse sarcoma [95]. Indeed, it has been shown that the microenvironment permits the survival of tumor cells without significant proliferation through a fine balance between antitumor immune cells and immunosuppressive cells [95]. A higher frequency of intratumoral cytotoxic T lymphocytes (CTLs), NK cells and γδ-T cells has been related to the maintenance of immune-mediated dormancy although the recruitment mechanism were not clarified.

### Keeping dormancy

Many studies revealed the importance of chronic inflammation in driving the reactivation of dormant cells [66, 96, 97]. However, context- and timing-dependent mixture of inflammatory cytokines, proteases, and stemness factors released by SnCs may stabilizes the senescence state in an autocrine manner, also affecting neighboring cells in a paracrine manner. Indeed, senescence can be maintained and reinforced by factors such as IL-1α and IL-6 whereas multiple SASP components, including TGF-β family ligands, VEGF, CCL2 and CCL20, can transmit non-cell autonomous growth arrest, referred to as paracrine senescence, to their neighbors [98, 99].

Intriguingly, in a recent paper, the authors through a study of integrating time-resolved gene expression and epigenomic profiles of cells escaping from oncogenic RAS-induced senescence in colorectal cancer, reveal that escape from OIS is accompanied by a gradual downregulation of SASP-associated genes and re-expression of genes that promote cell-cycle progression, confirming the role of a SASP-driven dormancy [70]. The excessive production of reactive oxygen species (ROS) by “dormant” senescent cells may also contribute to the maintenance of cellular senescence, via a positive self-amplifying loop, which constantly generates DNA damage [100]. Furthermore, a proinflammatory TME can promote macrophages to produce more ROS further amplifying this loop [101].

This evidence of a clinical-relevant dormant state of tumor cells comes from different in vitro and ex vivo models [102, 103], and from clinical data derived from late recurrence or relapse in cancer patients [104]. Nevertheless, the identity of dormant tumor cells remains elusive and studies directly identifying dormant tumor cells as senescent are scarce. However, the latest findings on the possibility of reversal of the senescent phenotype and the ability of senescent cells to release proinflammatory cytokines strongly support the idea that these cells are likely involved in cancer dormancy, escape, and recurrence.

## Senescence contribution to cancer progression: the “escape phase”

Different mechanisms of escaping immune control have been described in cancer progression mainly involving: (i) prevention of immune detection, (ii) prevention of immune activation and (iii) activation of immune suppression. Importantly, in the context of anti-cancer immune responses, these three mechanisms can occur simultaneously or in distinct phases.

### Senescence-driven mechanisms of escaping anticancer immune control

Induction of senescence and secretion of SASP can be regulated over the time during cancer progression, reshaping TME potentially promoting tumor growth and metastasis induction [105, 106] (Fig. 2B). In this regard, early SASP factors, such as TGF-β1 and -β3, can be immunosuppressive [107, 108] whilst the SASP secreted in a later time (deep senescence) often consists of an array of factors and proinflammatory cytokines, including the classical IL-1β, IL-6 and CXCL8, able to attract and activate a wide range of immune effector cells (e.g. CTLs, B-cells, neutrophils) [109], thus favoring the elimination of senescent cells, as previously described in the ‘‘senescence surveillance” process [110]. Noteworthy, in the case of IL-6, this cytokine can be also produced by dysregulated senescent stromal cells and has been shown to recruit myeloid suppressive cells to inhibit T-cell activation and response against malignant cells [111]. Moreover, an additional mechanism related to the persistent secretion of IL-6 by SnCs is the upregulation of HLA-E expression, able to impair their elimination by NK cells and CD8 + T cells via the inhibitory receptor NKG2A, thus inhibiting senescent cancer cell surveillance despite the presence of immune cell infiltrates [112]. A cogent example of this dual behavior of senescent cancer cells during progression has been provided by Eggert et.al [40]. where the authors described how, in HCC progression, senescence-induced immune surveillance requires the initial recruitment and maturation of CCR2^+^ myeloid cells; however, if SnCs are not cleared, fully established senescent HCC cells can repress the maturation of the recruited myeloid precursors, able to block the activity of NK cells and to promote tumor growth. This contradictory phenomenon can be explained by the plasticity of CCR2^+^ myeloid cells, upon entry into their end-organ tissue, depending on the microenvironment and cytokine milieu they can find [113, 114]. Noteworthy, senescence in peritumoral tissue of HCC patients has been associated with bad prognosis and survival of human HCC patients, and this was correlated with high CCL2 expression, accumulation of myeloid cells and low NK cell gene activity [40]. An additional example of the critical roles played by different microenvironment/cytokine milieu on myeloid cells regards macrophage polarization. Senescent hepatocytes/stellate cells have been shown to drive the polarization of macrophages toward an M1 phenotype, effective in containing liver tumor growth, through a SASP regulated by persistent p21 (Cdkn1a) expression [115]. In an opposite way, the activity of p53-deficient stellate cells, which cannot enter the senescent state, has been shown to produce a SASP able to promote M2 macrophage differentiation, thus contributing to the proliferation of premalignant tumor hepatic cells [36].

In obesity-related liver carcinoma, a different model of SASP-mediated immunosuppression, it was demonstrated that the cooperation between the hepatic translocation of the obesity-induced gut microbial component lipoteichoic acid (LTA), and the production of the obesity-induced gut microbial metabolite, deoxycholic acid, enhanced induction of SASP and the activity of COX2 in hepatic stellate cells (HSCs), by a Toll-like receptor 2 mechanism. Increased COX2-mediated prostaglandin E2 (PGE2) production suppressed anti-cancer immune response via the activity of the PTGER4 receptor, increasing formation of metastasis [116]. Thus, these findings broaden the molecular complexity associated with senescence-induced changes in the TME, highlighting that lipid mediators, such as PGs, are also produced by SnCs and act as immunomodulating SASP factors. In the context of TME, Krtolica and colleagues described for the first time that the phenomenon of cellular senescence occurs also in mesenchymal cells, revealing the important concept that cellular senescence, despite protecting against cancer in young adults, may promote cancer progression in aged organisms [106]. The SASP components released by senescent stroma include different cytokines and chemokines (e.g. CXCL8 and CXCL3), different growth factors, including amphiregulin (AREG), and are mainly involved in inducing and accelerating disease progression via paracrine actions [37, 105, 117, 118]. In particular, AREG, an epidermal growth factor (EGF) receptor ligand, is involved in the progression of many cancer types [119, 120]. Stromal cells induced to senescence by genotoxic treatments release AREG that confers increased resistance of prostate cancer cells against mitoxantrone, a DNA-targeting chemotherapeutic, and simultaneously induces the PD-L1 expression in tumors via a pathway that functionally involves EGFR and its downstream elements [121]. Cancer stage may also play a role in the ability of stromal SASP to influence tumor fate. SASP released from H_2_0_2_-treated mesenchymal stromal cells blocks proliferation and induces senescence of immortalized prostate cells while metastatic prostatic cancer cells appeared insensitive to SASP [122]. Furthermore, the immunomodulatory effect of senescent stroma is often correlated to the accumulation of MDSCs [23, 111].

Regarding the suppression of immune activating pathways as immune escape mechanism, it should be observed that persistent SnCs accumulate in cancer patients and are found in inflamed/damaged tissues, premalignant lesions and after chemo/radiotherapies [105, 123–125]. Here, the establishment of a senescence program in persistent SnCs has been shown to subvert immune clearance through the activity of MMP-dependent shedding of selected NKG2D-Ls and suppression of NKG2D-mediated immunosurveillance [126–128] leading to SnCs persistence, as described in cancer epithelial cells and in residual tumors from patients with breast and prostate cancer who underwent genotoxic chemotherapy [127]. Interestingly, the activity of p53 and p16 tumor suppressors was not required to induce or maintain the expression of NKG2D-Ls and MMPs in response to senescence-inducing genotoxic stresses after radiotherapy and chemotherapy [127]. Furthermore, the senescence-associated evasion process was restricted to genotoxic-resistant tumors, suggesting that a tailored senolytic therapy could eliminate deleterious resistant SnCs, avoiding possible side effects. Here, the elimination of persistent SnCs by pharmacological strategies has shown beneficial effects in mouse models [129–131] and approaches designed to boost the immune recognition of persistent SnCs could represent a promising strategy. These aspects will be further discussed.

### Immunosenescence as further player in the “escape phase”

Immunosenescence is defined as a dysregulation of immune cell function that contributes to the increased susceptibility to several age-related diseases (including cancer). However, the senescence of the immune system can also occur independently by the age. Indeed, the accumulation of dysfunctional senescent T-cell types have been found in relatively young patients with certain types of cancers, including lung cancers, colorectal cancer, head and neck cancer, ovarian cancer and hepatocellular carcinoma [132–137]. In this regard, malignant tumors can promote T cell exhaustion and senescence leading to a defective effector function in the TME [136–138]. These two important dysfunctional states that share overlapping characteristics, mostly the loss of proliferative potential and CD28 surface expression [139], favor cancer progression. What is currently widely recognized as a major distinguishing difference is that exhausted T cells are characterized by elevated expression of a panel of inhibitory receptors (e.g. PD-1, CTLA-4, TIM-3) [140–142] while senescent T cells, together with the high expression of SA-β-Gal [143–145], dramatically upregulate additional senescence-associated markers, including CD57, CD45RA, and killer cell lectin-like receptor subfamily G member 1 (KLRG-1) [146, 147]. In addition, unlike exhausted T cells, senescent T cells present decreased expression of functional molecules related to cytotoxic activity, such as perforin and granzyme B, in vivo [148], and acquire a particular SASP, generating elevated amounts of proinflammatory and strongly suppressive cytokines, such as IL-10 and TGF-β [143] that contribute to the functionally hyporesponsive states of the immune system observed in the TME during cancer progression. In addition to this, in recent years, several studies have demonstrated that tumor-associated Treg cells can directly induce T cell senescence in the TME [149, 150]. Indeed, naturally occurring human CD4^+^CD25^hi^FoxP3^+^ Treg cells can selectively modulate p38 and ERK1/2 MAPK signaling pathways, suppressing T cell proliferation through induction of senescence on responding naive and effector T cells [143]. Treg cells also display predominant dependence on glucose metabolism compared with effector T cells thus increasing the glucose consumption that leads to cell senescence and suppression in responder T cells [150]. Interestingly, the TLR8 signaling selectively inhibits glucose uptake resulting in reversal of Treg suppression opening the possibility of identifying new cancer treatment strategies [151]. In *vitro* studies on human T cells have also found that induction of the DNA damage response, caused by metabolic competition, is the main cause of Treg-mediated induction of senescence on responder T cells. Indeed, the authors demonstrated that the stress-induced ERK1/2 and p38 MAPK signaling pathways functionally cooperating with the STAT1/STAT3 transcription factors, can control Treg-induced T-cell senescence [149].

Some characteristics of senescence in human effector memory CD8^+^ T cells that re-express the naive T cell marker CD45RA (EMRA), such as mitochondrial disruption and ROS accumulation are mediated by p38 MAPK signaling and are reversible [152]. In this scenario, the possibility to revert the senescent CD8^+^ phenotype is important, as the number of EMRA CD8^+^ T cells increases in metastatic melanoma patients [153].

Altogether these studies suggest that T-cell senescence is an alternative novel mechanism utilized by malignant tumors for immune evasion and provide the rationale for tumor immunotherapy. Finally, an additional layer of complexity is the observation that, during aging, there is an accumulation of CD8^+^ T cells with the senescence phenotype that acquire NK function, thus compensating for reduced TCR-mediated classical T cell function and involved in tumor elimination [154]. Indeed, CD27^−^CD28^−^CD8^+^ T cells with the senescence phenotype can acquire a NK function upregulating the expression of NKRs such as NKG2D, NKG2A and CD16 and are involved in tumor elimination in *vitro* and in *vivo* [155]. Accordingly, these observations have been confirmed in a B16 melanoma lung metastasis murine model, where it has been demonstrated that Menin-deficient senescent CD8^+^ T cells from aged mice [156, 157], play a role in antitumor immunity by acquiring NK cell-like innate immune functions [158]. Nonetheless, the role of senescent CD8^+^ T cells in tumor immunity remains to be fully elucidated.

## Tracking the dynamics of cellular senescence via the cGAS/STING axis

**Fig. 3:**
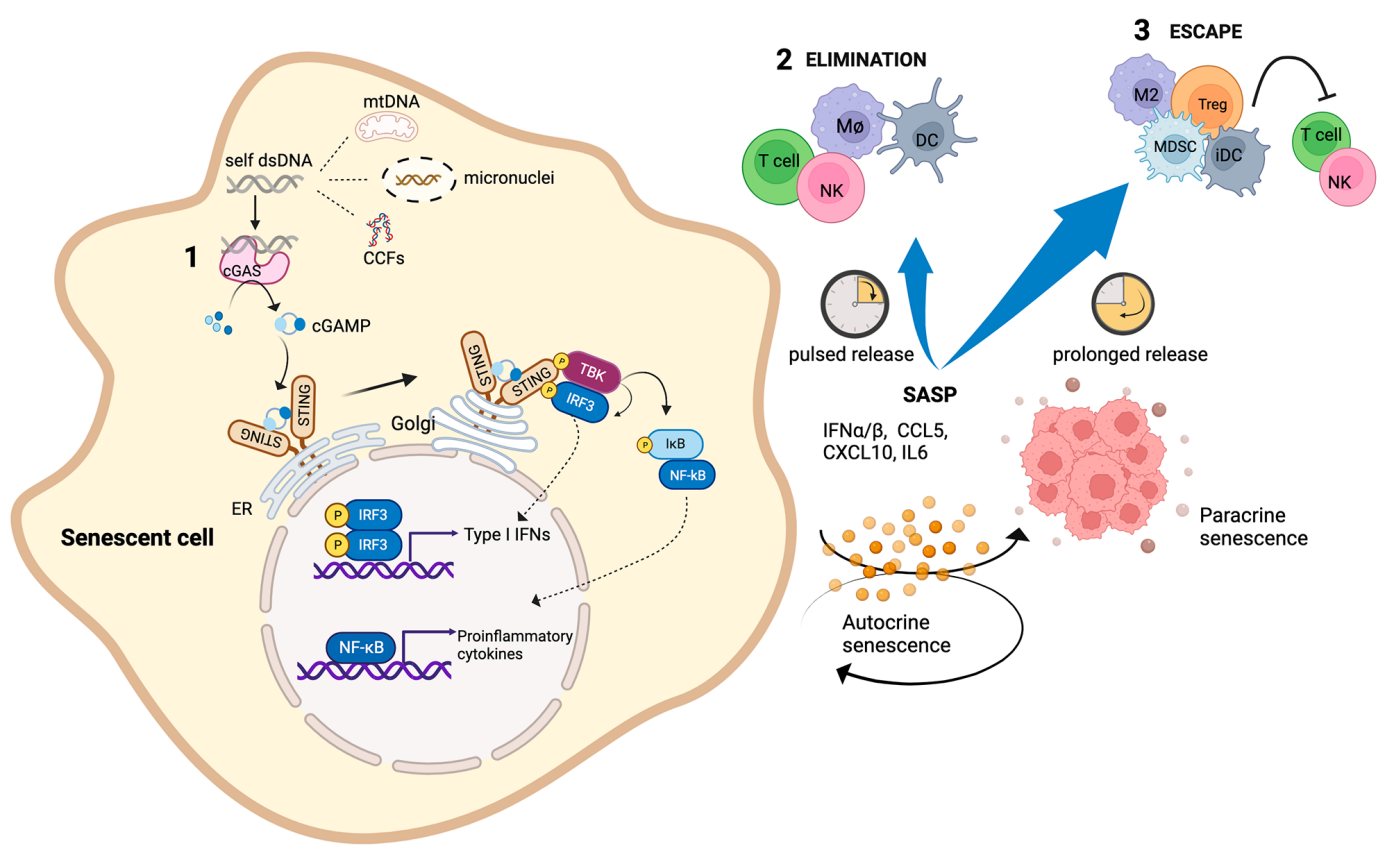
cGAS/STING pathway as driver of senescence and immunosurveillance (**1**) Upon binding to cytosolic dsDNA, cGAS drives the production of cGAMP that is detected by STING on ER membrane. Then, STING transfers to the Golgi apparatus and recruits TBK1 and IκB kinases to activate IRF3 and NF-κB, respectively, giving rise to the production of type I IFNs and a plethora of inflammatory cytokines and chemokines that are secreted in the extracellular milieu representing the SASP components. (**2**) SASP factors promote autocrine and paracrine senescence, moreover they contribute to immune cell recruitment and activation favoring the elimination of cancer senescent cells. (**3**) Sustained cGAS/STING activation over time, following accumulation of cytosolic dsDNA in senescent cancer cells, determines the persistence of a pro-inflammatory and pro-tumoral SASP favoring the generation of immunosuppressive populations that negatively affect cancer immunosurveillance. Abbreviations: Treg (T regulatory cells); MDSC (Myeloid Suppressor Cells); iDC (immature Dendritic Cells) Created with BioRender.com with granted permission and license

In the last few years, endogenous DNA sensing through the cGAS/STING pathway has emerged as an important regulator of senescence and SASP. In this regard, the relationship between the cGAS/STING axis and senescence can be viewed as an example of the complex crosstalk between senescence induction and anti-cancer immune response taking into consideration its contribution to cancer “immunoediting”. Accumulation of double strand DNA (dsDNA) in the cytoplasm of senescent cancer cells leads to the activation of the stress sensor pathway mediated by the cyclic guanosine monophosphate-adenosine monophosphate (cGAMP) synthase (cGAS) and the adaptor stimulator of interferon genes (STING). Upon binding to dsDNA, cGAS drives the production of cGAMP, a second messenger detected by STING on the endoplasmic reticulum (ER) membrane. After that, STING transfers to the Golgi apparatus and recruits TANK-binding kinase 1 (TBK1) and IκB kinase to activate IFN regulatory factor 3 (IRF3) and NF-κB, respectively, giving rise to the production of type I interferons (IFNs) and inflammatory cytokines and chemokines [159, 160] (Fig. 3).

Senescent cells are characterized from the presence of CCFs, micronuclei and mitochondrial DNA (mtDNA) that represent important sources of immunostimulatory self-DNA able to activate the cGAS/STING pathway, thereby regulating the SASP and facilitating paracrine senescence [161–164]. Gluck and colleagues found that diverse triggers of cellular senescence, including oxidative stress, oncogene signaling, irradiation, and pro-senescent drugs (i.e.: palbociclib) depend on cGAS/STING signaling to drive the production of several SASP components [162]. As such, mouse embryonic fibroblasts deficient in cGAS exhibited reduced signs of senescence [165] and defects in cGAS or STING alter SASP composition [166]. In line with these evidences, Omer and colleagues reported that genetic depletion or chemical inhibition of the factor Ras GTPase activating protein-binding protein 1 (G3BP1) which impairs the association of cGAS with cytoplasmic DNA, prevents the expression of SASP factors without affecting cell commitment to senescence [167, 168].

As stated above, SASP is a key feature of senescent cells that mainly acts to reinforce cellular senescence in a non-cell autonomous manner. Through the auto and paracrine effects of the SASP, senescent cells modulate their own cellular fate and influence the surrounding microenvironment including immune cell functions and their recruitment. For instance, type I IFNs strongly contribute to the generation of a senescent phenotype [169, 170]. In particular, prolonged exposure to IFN-β induced a p53-dependent cellular senescence program in normal human fibroblasts [169]. The importance of cGAS-STING pathway-induced SASP and its role in cancer immune surveillance has been extensively described in several experimental models. For instance, in an in vivo model of OIS, Dou and colleagues have demonstrated that STING is essential for RAS-induced SASP and for the immune-mediated clearance of cancer cells [161]. Similarly, in an ovarian tumor model, the induction of SASP mediated by the GAS/STING pathway was shown to be decisive in promoting T cell recruitment into tumors [171]. Also, selective recruitment and activation of CD8^+^ T cells into mismatch repair-deficient colorectal cancers strictly depends on CCL5 and CXCL10 chemokines due to endogenous activation of cGAS/STING and type I IFN signaling by damaged DNA [172]. Another consideration to be made concerns the production of the second messenger cGAMP by tumor cells and its diffusion in the tumor microenvironment with consequent activation of STING in other cell types. Accordingly, it has been shown that cancer cell intrinsic expression of cGAS is critical for cancer immune surveillance since cGAMP produced by cancer cells is transferred to surrounding DCs and promotes type I IFN production in a STING dependent fashion [173].

In this scenario, the cGAS/STING pathway contributes to the elimination phase acting as a bridge connecting innate and adaptive immune response through an immunostimulatory SASP. On the other hand, continuous cGAS/STING activation over time, following accumulation of cytosolic dsDNA in senescent cancer cells, determines the persistence of a pro-inflammatory and pro-tumoral SASP favoring the generation of immunosuppressive populations that negatively affect cancer immunosurveillance (Fig. 3).

In addition to this, it is also important to highlight the recurrent inactivation of cGAS/STING pathway in a wide variety of cancers [163, 165, 174, 175] suggesting a potential additional immune escape mechanism driven by modulation of the cGAS/STING pathway. Nonetheless, the impact of cGAS/STING inactivation in the crosstalk between senescence and cancer immune surveillance remains to be fully elucidated.

Overall, these studies emphasize multifaceted roles of cGAS/STING pathway in the induction, maintenance and persistence of senescence and its remarkable influence on immune cell response through SASP-related components. Therefore, it will be of interest to extend the field of application of drugs targeting cGAS or STING developed for cancer immunotherapy [176] as novel regulators of senescence cell fate.

## Modulation of senescence for cancer immunotherapy

Accumulation and persistence of SnCs in cancer patients has been proven to be detrimental to the organism. Selective removal of senescent cells, namely senotherapy, has been proposed as a promising adjuvant approach to eliminate the adverse effects of SnCs [177–179]. Senotherapy can be pursued through different strategies including: (i) the usage of senolytics, i.e. drugs targeting senescent cells; (ii) modulation or inhibition of SASP (senomorphics); (iii) immune cell-mediated clearance of senescent cells. The use of senotherapy in combination with current cancer therapies represents the so-called “one-two punch” approach in which cancer cells are forced to senescence by pro-senescence therapies and then killed by senolytic drugs or by the action of the immune system. Albeit metabolically active, senescent cells are cell cycle arrested and, therefore, cellular senescence has been viewed as a desirable outcome during cancer treatment, provided that SASP adverse effects are abrogated by senomorphics. Herein we will focus above all on approaches aimed at enhancing the recognition and elimination of SnCs by immune cells.

### Senolytic drugs and senomorphics

Briefly, the development of senolytic drugs is mainly based on the inhibition of biological pathways essential for the survival of senescent cells. This includes the targeting of the BCL-2 family members, p53/p21 axis, PI3K/AKT pathway, receptor tyrosine kinases, and HIF-1α and HSP90 proteins. For instance, Navitoclax/ABT-263 or ABT737 compounds target BCL-2 family members and are effective to induce cell death in SnCs. However, these compounds have been shown to be highly harmful acting also on non-SnCs [180]. In a different way, HSP90 inhibitors, recently identified as new class of senolytic agents in a drug screening approach, are immunomodulators with potent anti-tumor activity [181]. In this regard we and others have demonstrated that HSP90 inhibitors induce the expression of NKG2D ligands on cancer cells that become more sensitive to NK cell mediated attack [182, 183].

Novel therapeutic strategies aimed at preventing the detrimental activity of SnCs are focused on the inhibition or modulation of SASP without killing SnCs by use of the so-called “senomorphics”. Distinct approaches have been carried out so far to reprogram or abrogate SASP that is regulated at epigenetic, transcriptional and post-transcriptional levels. The majority of senomorphics target key SASP regulators including chromatin readers (i.e.: BRD4, MLL1, HMGB2), JAK2/STAT3, cGAS/STING, mTOR, p38MAPK, and NF-kB [12, 177–179].

A large body of evidence has described the importance of epigenetic regulation in the control of SASP. As such, inhibition of the BET protein BRD4 reduced the production of IL-1α, IL-1β, IL-6, and CXCL8 in senescent fibroblasts [42]. In addition, suppression of other chromatin modifiers including mixed-lineage leukemia protein 1 (MLL1) and high-mobility group box 2 (HMGB2) [184, 185] hampered SASP inflammatory factors without altering the senescence phenotype.

Through an RNAi screen, the splicing factor polypyrimidine tract binding protein 1 (PTBP1) was identified as potent SASP modulator as its depletion suppresses NF-kB pathway and blunts pro-inflammatory cytokine secretion in various cell types exposed to different senescence-inducing stimuli. Moreover, PTBP1 knockout mice in a model of liver cancer, decreased immune cell infiltrate [186]. Pharmacological inhibition of the JAK2/STAT3 pathway in *Pten*-deficient senescent prostate tumors modified SASP composition, leading to an anti-tumor immune response that enhances chemotherapy efficacy [187]. The role of JAK/STAT pathway in the modulation of SASP was further explored in senescent preadipocytes and umbilical vein endothelial cells (HUVEC) [188]. It was discovered that different JAK inhibitors reduced the production of some cytokines including IL-6, CXCL8, CCL2, CXCL1 and in vivo administration of the JAK1/2 inhibitor ruxolitinib reduced systemic and adipose tissue inflammation in aged mice [188].

As already mentioned, the cGAS-STING pathway plays a central role in the establishment of senescence and SASP. For instance, G3BP1 depletion or its pharmacological inhibition impairs the cGAS-pathway preventing the expression of SASP factors (i.e.: IL-6, TNFα, IFNβ, CXCL8) without affecting the senescence phenotype [168]. Thus, the usage of cGAS or STING inhibitors could represent an alternative approach to modulate the release of SASP components avoiding their accumulation over time [189] (Fig. 3).

In a drug screen to find novel SASP regulators, rapamycin, a selective inhibitor of the mTOR complex 1 (mTORC1), abrogated the induction of a number of SASP components through a mechanism dependent on downregulation of mitogen-activated protein kinase-activated protein kinase 2 (MAP-KAPK2 or MK2), a downstream effector of p38, thereby reducing breast tumor growth in mice [190]. It should be noted that mTOR can affect SASP at several levels; an elegant study by Laberge and colleagues reported that rapamycin modulates SASP by reducing *IL-1*α mRNA translation and as consequence IL1R1 signaling leading to a decreased NF-κB transcriptional activity. In this context, the NF-κB-dependent expression of IL-6 and CXCL8 was suppressed in senescent cells [191]. As matter of fact, in a model of OIS into human lung embryonic fibroblasts, *IL-1a* knockdown was shown to weaken the late proinflammatory arm of SASP controlled by NF-κB without altering cell cycle arrest [192]. Moreover, ablation of the SASP through the deletion of *IL-1α* gene in a mouse model of pancreatic cancer impairs tumor progression [192].

Based on the above findings, selective removal of one or more SASP components may represent a promising strategy to reduce SASP detrimental effects since monoclonal antibodies and other agents targeting inflammatory cytokines and their receptors are clinically approved for inflammatory disorders. These include drugs targeting IL-1α/IL-1β/IL1R (Anakinra, Canakinumab, Rilonacept) [193, 194], TNFα (Infliximab, Etanercept) [195], and IL-6/IL-6R (Tocilizumab, Siltuximab) [196]. Interestingly, new nanotechnologies settled on the employment of galactose-modified nanoparticles have been recently proposed to increase the specificity of drug delivery into senescent cells [197, 198]. Table 1 recapitulates some experimental models of senescence used to study SASP modulation.

**Table 1:** Targeting SASP regulators in experimental models of senescence

Experimental model of senescence	Cell type	SASP regulators	Pharmacological inhibition	Genetic inhibition	Reference
H-RAS ^V12^	Human fibrobasts (IMR90)	HMGB2	Nd	shRNA	[184]
4-OHT/H-RAS^V12^	Human fibrobasts (IMR90)	MLL1	M-2-2	shRNA	[185]
H-RAS ^V12^	Human fibrobasts (IMR90; WI-38)	BRD4	JQ1	shRNA	[42]
Pten^−/−^ mice	Prostate tumors	JAK2/STAT3	NVP-BSK805	Stat3^−/−^ mice	[187]
Ionizing radiation	Pre-adipocytes; HUVEC	JAK1/2	Ruxolitinib	nd	[188]
Ionizing radiation	Human fibrobasts (IMR90 and WI-38)	G3BP1	EGCG*	shRNA	[168]
4-OHT/ ER:RAS	Human fibrobasts (IMR90; BJ;HFFF2)	mTORC1	Rapamycin Torin1 NVP-BEZ235	shRNA	[190]
4-OHTor Etoposide/ H-RAS^V12^; pancreatic cancer mouse model	Human fibrobasts (IMR90)	IL1R/IL-1α	Nd	shRNA; IL1R^−/−^ mice	[192]
4-OHT/ ER:RAS	Human fibrobasts (IMR90);MR90;MCF7	PTBP1	Nd	siRNA	[186]

Abbreviations: *Epigallocatechin gallate (EGCG)

Studies on senomorphics still lack of completeness, as most of the research has largely employed senescence models derived from tumor suppressors loss of function (i.e. *p53, PTEN*) or from OIS. It is not supposed that similar results could be obtained in models of TIS. Additional work is urgently needed to elucidate how modulation of SASP in different model of senescence and in different cancer contexts may impact tumor immunosurveillance.

### Harnessing the immune cell mediated clearance of SnCs

As previously discussed, NK cells strongly contribute to the elimination of senescent cells in different cancer models, thus NK cell engaging therapies such as therapeutic antibodies or adoptive NK cell transfer may further increase the clearance of SnCs [199]. Indeed, combined high-dose radiotherapy and adoptive NK cell transfer improve tumor control over monotherapies in xenografted mice. Noteworthy, the authors showed that CXCL8, as a SASP component, was responsible for NK cell recruitment and infiltration to the tumor site [200]. In a different therapeutic strategy, targeting the inhibitory receptor NKG2A that binds the non-classical MHC-class Ib molecule HLA-E, could be employed as a senolytic strategy to boost NK cell activity. As stated before, HLA-E was found upregulated on the cell surface of senescent fibroblasts exposed to the SASP-related cytokine IL-6. Inhibition of HLA-E expression through RNA interference enhanced NK cell-mediated cytotoxicity against SnCs [112] suggesting the usage of monalizumab, a therapeutic antibody targeting NKG2A, in cancer patients [201].

An additional relevant therapeutic target to consider derived from the observation that SnCs can evade NK cell mediated surveillance through the shedding of NKG2DLs [126–128]. Hence, strategies aimed at stabilizing NKG2DLs on the cell surface of SnCs look promising to foster NK cell senescence surveillance. For instance, antibodies blocking MICA/B protease-mediated cleavage have been developed and their effectiveness has been assessed in pre-clinical models [202]. In another study, Zingoni and coworkers demonstrated that the combined use of chemotherapeutic drugs and metalloproteinase inhibitors strengthened NK cell-mediated killing of senescent multiple myeloma cells, preserving the expression of MIC molecules on the cell surface [126].

A fast-developing field in cancer immunotherapy relies on the search of cell surface proteins specifically or highly expressed by SnCs that can be employed to design antibodies and chimeric antigen receptors (CAR). Of interest, by mass spectrometry analysis, dipeptidyl peptidase 4 (DPP4 or CD26) was identified as a surface marker of senescent human fibroblasts. Moreover, DPP4^+^ SnCs opsonized with an anti-DPP4 specific antibody were handled as NK cell targets in an antibody-dependent cell-mediated cytotoxicity (ADCC) assay [203].

Amor and colleagues identified the urokinase-type plasminogen activator receptor (uPAR) as a SnC-surface marker and showed that uPAR-specific CAR T cells can efficiently eliminate lung cancer cells induced into senescence in response to combined therapeutic treatment (i.e. MEK plus CDK4/6 inhibitors). Further, in a xenograft model of lung cancer, uPAR-specific CAR T cells selectively killed SnCs in tumor tissues and extended the survival of mice bearing lung adenocarcinoma [204]. Interestingly, as already mentioned, NKG2DLs are highly expressed by SnCs and have been proposed as target molecules for CAR T cell therapy. A recent study demonstrated that NKG2D-CAR T cells recognized and killed NKG2DL^+^ SnCs in aged mice. Furthermore, the use of murine NKG2D-CAR T cells in two independent mouse models of aging, reversed senescence-associated phenotypes, highlighting their potential in treating aging and age-related diseases, including cancer [205]. These reports indicate that T-CAR technology applied to the recognition of senescent tumor cells could boost therapies based on the induction of senescence. Novel approaches aimed at eliminating dysfunctional senescent T lymphocytes may also contribute to enhance cancer immunosurveillance [149, 151]. For example, a vaccine targeting CD153 has been found to deplete senescent T lymphocytes from high-fat diet-induced obese mice [206].

### Targeting the immune checkpoint inhibitors (ICi) PD-1 and PDL-1/2

A large body of evidence has described the upregulation of the immune checkpoint molecule PD-L1 [58, 207–209] and PD-L2 [210] in SnCs leading to an impairment of T cell mediated immune response.

Accordingly, PD-L1 targeting combined with chemotherapy could be effective to potentiate the clearance of SnCs. In this regard, a recent study reported that the combined use of PARPi (talazoparib) and CDK4/6i (palbociclib) potently triggered TIS in colorectal cancer cells and promoted T cell-mediated anti-tumor immune response. In this context, characterization of SASP components revealed the presence of type I IFNs and some interferon-stimulated genes (ISGs) like CCL5 and CXCL10 chemokines as result of the activation of cGAS/STING signaling pathway. The addition of the immune checkpoint inhibitor anti-PD-L1 further improved the clearance of senescent cancer cells in immunocompetent mice [209]. In a *Trp53* WT mouse mammary tumor model, chemotherapy followed by treatment with anti-PD-L1, stimulated a substantial accumulation of T cells within the tumor and a concomitant anti-tumor immune response. In addition, single cell analysis data showed that senescent PD-L1^+^ cells are characterized by enhanced IFNγ signaling (*Stat1*, *Irf1*, and *Lgals9*) and display higher PD-L1 expression compared to non-senescent cells [58]. Accordingly, upregulation of PD-L1 expression was previously shown to be regulated by both type I and type II interferons [211] and it is interesting to note that independently from the trigger (i.e.: replication exhaustion, activated oncogene HRasV12, DNA-damaging agent etoposide, or ionizing irradiation), PD-L1 is upregulated in all forms of senescence in primary human diploid lung fibroblasts [208].

Several studies provided evidence that secretome produced by senescent cells could contribute to the recruitment of PD-1^+^ T lymphocytes within the tumor. Remarkably, using a pre-clinical mouse model of breast cancer brain metastasis (BCBM), Uceda-Castro and colleagues discovered that doxorubicin-induced senescence in BCBM tumor cells triggers a significant recruitment of PD-1^+^ CD8^+^ T lymphocytes to BCBM lesions thus promoting the efficacy of anti-PD-1 treatment [212].

Moreover, in a preclinical mouse model of KRAS-mutant pancreatic ductal adenocarcinoma, chemotherapy-induced senescent cancer cells led to the secretion of pro-angiogenic SASP factors, which favored the intratumoral accumulation of CD8^+^ T cells, thereby sensitizing tumors to PD-1 checkpoint blockade [45]. Taken together these studies highlight how anticancer immunotherapies combined with ICi reinforce the efficacy of senotherapy as well as its outcome.

**Fig. 4:**
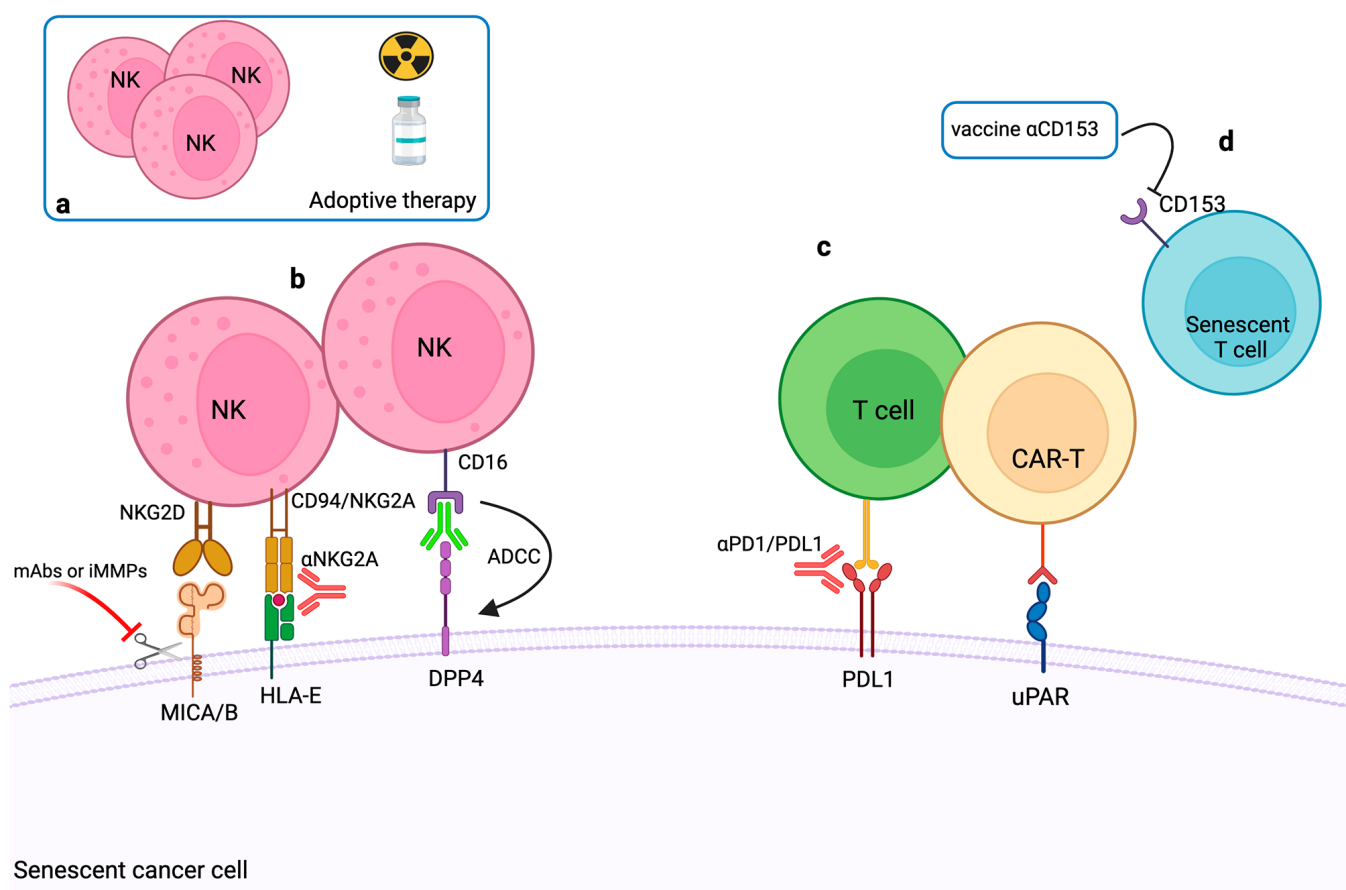
Immune cell-mediated clearance of senescent cells **a**) Adoptive NK cell transfer combined with radio and chemotherapy. **b**) Approaches aimed at fostering NK cell recognition and killing of senescent cells including: (i) stabilization of NKG2DLs on senescent cell surface by blocking the protease-mediated cleavage through iMMPs or mAbs targeting the MICA/B shedding domain; (ii) targeting CD94/NKG2A inhibitory receptor using anti-NKG2A therapeutic mAb (i.e.: monalizumab); (iii) ADCC triggered by anti-DDP4 mAb. **c**) Enhancement of T cell mediated functions by targeting PD-1/PDL-1 axis; generation of uPAR specific CAR-T cells; **d**) depletion of senescent T lymphocytes following αCD153 vaccination Abbreviations: iMMPs (metalloproteinase inhibitors); ADCC (Antibody dependent cellular cytotoxicity); NKG2DLs (NKG2D ligands) Created with BioRender.com with granted permission and license

In Fig. 4, immunotherapeutic strategies involved in the clearance of SnCs are illustrated.

## Conclusions

Cancer immunoediting theory represents the conceptualization of the complex and highly dynamic interaction that exists between neoplastic growth and the immune system. Unfortunately, some pieces of this picture are missing. Cellular senescence is one of this, as its impact on tumor progression is still unclear. Initially described as a defense mechanism against malignant transformation, its contribution to inflammation can definitely favor cancer progression. In both cases cellular senescence has an intimate connection with the immune system. As debated in the present review, there is strong evidence of the role of senescence in shaping the elimination and escape phases of cancer immunoediting, with implications that should not be neglected in tailoring the upcoming therapeutic strategies. The equilibrium phase, instead, has been less investigated so far due to the lack of experimental models and technical difficulties, but the discovery of dormant cancer cells and the new methodologies about single cell profiling are shedding light on this aspect so relevant for the biology of tumors. In this context, the connection between dormancy and senescence is extremely interesting and approaches currently used for targeting SnCs could be extended to dormant cancer cells. It should be noted that findings derived from different experimental models or settings could not drive to similar conclusions. A factor of complexity is given by the high variable spectrum of immune responses which characterize different types of tumors, spanning from hematological to solid tumors or slow to fast growing cancers. Furthermore, the senescent phenotype can affect not only cancer cells but also cells of the TME impacting on the mechanisms of tumor growth (e.g. epithelial to mesenchymal transition) and drug resistance. SnCs are nowadays considered a key component of premalignant lesions and tumor mass due to oncogenic and other cancer-related stresses, where they can act either as antitumor barrier or inflammatory agent, as stated above. Relevant for anticancer therapies, senescence is a common outcome of different genotoxic drugs and molecularly targeted agents routinely adopted in clinical settings, with uncertain effects on therapy efficacy. Senescence may serve as one mechanism of drug resistance and tumor dormancy, thus contributing to cancer relapse, as senescent cells share characteristics with dormant cancer cells. For all these reasons, cellular senescence deeply affects cancer immunoediting all along the way, pushing the action of the immune system into even opposite directions. Uncovering the underlying mechanisms that link senescence to cancer immunoediting is thus mandatory to depict the complex immune landscape that characterizes tumor progression and consequently adopt successful actions.

## Data Availability

No datasets were generated or analysed during the current study.

## Abbreviations

ADCC: Antibody Dependent Cellular Cytotoxicity
AREG: Aphiregulin
BET: Bromo and Extra Terminal domain
CAR: Chimeric Antigen Receptor
cGAS: Cyclic GMP-AMP Synthase
CCFs: Cytoplasmic Chromatin Fragments
CTLs: Cytotoxic T lymphocytes
DDR: DNA Damage Response
dsDNA: Double Strand DNA
EGCG: Epigallocatechin gallate
EGF: Epidermal Growth Factor
ER: Endoplasmic Reticulum
HCC: Hepatocellular Carcinoma
HPCs: Hepatic Stellate Cells
ICi: Immune Check Point Inhibitors
ISGs: Interferon Stimulated Genes
KLRG-1: Killer Cell Lectin-Like Receptor Subfamily G Member 1
LTA: Lipoteichoic Acid
MM: Multiple Myeloma
MDSC: Myeloid-derived suppressor cells
NK: Natural Killer
NKG2DL: NKG2D Ligand
OIS: Oncogene-Induced Senescence
PGE2: Prostaglandin E2
ROS: Reactive Oxygen Species
SASP: Senescence-Associated Secretory Phenotype
SnCs: Senescent Cancer Cells
STING: Stimulator of Interferon Genes
TBK: TANK-Binding Kinase
TILs: Tumor-Infiltrating Lymphocytes
TIS: Therapy-Induced Senescence
TME: Tumor Microenvironment
TNA: Tumor Neoantigens
TRM: Tissue resident memory cells
